# Five-year prognostic impact of pre-existing interstitial lung disease in non-small cell lung cancer patients treated with anti-PD-1 monotherapy: a retrospective analysis

**DOI:** 10.1186/s12885-026-15989-1

**Published:** 2026-04-25

**Authors:** Teppei Yamaguchi, Junichi Shimizu, Reiko Matsuzawa, Kazuhiro Shimomura, Naohiro Watanabe, Yoshitsugu Horio, Yutaka Fujiwara

**Affiliations:** 1 Department of Thoracic Oncology, Aichi Cancer Center Hospital, 1-1, Kanokoden, Chikusa-ku, Nagoya, Aichi 464-8681 Japan; 2 Department of Respiratory Medicine, Nagoya University Graduate School of Medicine, 65 Tsurumai-cho, Showak-ku, Nagoya, Aichi 466-0065 Japan; 3 Department of Pharmacy, Aichi Cancer Center Hospital, 1-1, Kanokoden, Chikusa-ku, Nagoya, Aichi 464-8681 Japan

**Keywords:** Non-small cell lung cancer, Pneumonitis, Immune checkpoint inhibitors, Interstitial lung disease

## Abstract

**Background:**

Anti-programmed death 1 (PD-1) antibodies, a type of immune checkpoint inhibitor (ICI), have enhanced survival rates in non-small cell lung cancer (NSCLC). While some patients with NSCLC have pre-existing interstitial lung disease (ILD), data on the long-term safety and effectiveness of anti-PD-1 antibodies for this group are still unclear.

**Methods:**

We examined patients with advanced NSCLC who experienced recurrence after surgery or radiotherapy and received anti-PD-1 antibody monotherapy at Aichi Cancer Center Hospital from December 2015 to December 2018. The study assessed the connection between progression-free survival (PFS) and overall survival (OS) with respect to pre-existing ILD, and also investigated how ICI-induced pneumonitis impacted OS.

**Results:**

Among the 200 patients, 35 (17.5%) had pre-existing ILD. There were no significant differences in OS between the ILD and non-ILD groups, with 5-year OS rates of 21.1% and 24.8% respectively (HR = 0.99; 95% CI, 0.66–1.48; *p* = 0.956). ICI-induced pneumonitis was more common in the pre-existing ILD group (40.0% vs. 12.1%, *P* < 0.001). A 90-day landmark analysis showed no statistically significant difference in OS between patients with and without ICI-induced pneumonitis.

**Conclusion:**

Although the occurrence of ICI-induced pneumonitis was common, patients with NSCLC having pre-existing ILD exhibited comparable long-term survival rates to those without ILD. The potential risks and benefits should be thoroughly assessed when contemplating ICIs for these patients.

**Supplementary Information:**

The online version contains supplementary material available at 10.1186/s12885-026-15989-1.

## Background

Lung cancer is the leading cause of cancer-related death worldwide, with a 5-year survival rate of only 10%–20% [1]. Anti-programmed death 1 (PD-1) antibodies, a type of immune checkpoint inhibitor (ICI) designed to leverage the immune system to target and destroy cancer cells, have revolutionized treatment [2–5].

Interstitial lung disease (ILD) is a pulmonary condition marked by inflammation and/or fibrosis of the lungs’ parenchyma [6]. Among idiopathic ILDs, idiopathic pulmonary fibrosis has been explicitly linked to an increased risk of developing lung cancer [7–9]. Conversely, approximately 10% of patients with non-small cell lung cancer (NSCLC) are also found to have pre-existing ILD at diagnosis [10, 11].

Anti-PD-1 antibodies enhanced the prognosis for patients with NSCLC in several phase III clinical trials, particularly when actionable driver mutations are absent [2–5]. For example, a pooled analysis of the CheckMate-017 and CheckMate-057 trials showed that the 5-year survival rate was 13.4% for patients treated with nivolumab, compared to 2.6% for those treated with docetaxel in previously treated NSCLC (HR = 0.68; 95% CI, 0.59–0.78) [2, 3, 12]. The improvement in prognosis is especially notable in cases with programmed death-ligand 1 (PD-L1) expression of 50% or higher. In the Keynote-024 trial, pembrolizumab alone resulted in a median OS of 26.3 months, compared to 13.4 months with chemotherapy in treatment-naïve stage IV patients with NSCLC (HR = 0.62, 95% CI, 0.48–0.81) [4, 13]. The 5-year overall survival (OS) rates were 31.9% for the pembrolizumab group and 16.3% for the chemotherapy group. However, the survival benefit of ICIs for patients with NSCLC having pre-existing ILD remains uncertain, as most clinical trials excluded these patients due to their higher risk of ICI-induced pneumonitis. A small, single-arm phase II study showed that nivolumab can be effective in patients with NSCLC and pre-existing ILD, but this study only reported short-term outcomes [14].

Pre-existing ILD is associated with a higher risk of developing ICI-induced pneumonitis [15, 16]. Despite the increased risk, some patients with pre-existing ILD experience significant clinical benefits from anti-PD-1 antibodies, such as long-lasting responses and prolonged survival. There are few reports on the long-term outlook for patients with NSCLC and pre-existing ILD who have received anti-PD-1 therapy. Nonetheless, such data are crucial for understanding the balance between potential risks and therapeutic advantages. Therefore, this retrospective study was conducted to examine the long-term prognosis of patients with NSCLC and pre-existing ILD treated with anti-PD-1 antibodies.

## Methods

### Patients

We retrospectively analyzed patients with advanced NSCLC who were unresectable or recurrent after surgery or radiotherapy and began anti-PD-1 antibody monotherapy as their initial ICI treatment at Aichi Cancer Center Hospital between December 2015 and December 2018. Eligible patients had an Eastern Cooperative Oncology Group performance status (PS) of 0–2. Exclusion criteria included patients with epidermal growth factor receptor mutations, anaplastic lymphoma kinase rearrangements, or ROS proto-oncogene 1 rearrangements; patients who had participated in clinical trials receiving nivolumab or pembrolizumab; patients without an available baseline CT scan prior to study entry; and those with evidence of active pneumonia on baseline CT scan, which could interfere with imaging assessments. The presence of pre-existing ILD was determined using chest helical computed tomography (CT) scans taken before starting anti-PD-1 monotherapy. Baseline chest CT findings were reviewed by two pulmonologists (JS, RM) according to clinical practice guidelines jointly published by the American Thoracic Society, the European Respiratory Society, the Japanese Respiratory Society, and the Latin American Thoracic Association, with any discrepancies resolved through consensus discussions. Interstitial lung disease patterns on baseline chest CT were classified into the following categories: usual interstitial pneumonia (UIP) pattern, probable UIP, indeterminate for UIP, or an alternative diagnosis. ICI-induced pneumonitis was classified based on the National Cancer Institute Common Terminology Criteria for Adverse Events, version 5.0. PD-L1 expression was evaluated using either the Dako PD-L1 immunohistochemistry (IHC) 22C3 pharmDx assay (Agilent Technologies, Santa Clara, California, USA) or the Dako PD-L1 IHC 28 − 8 pharmDx assay (Agilent Technologies, Santa Clara, California, USA); if both results were available, the 22C3 assay result was used.

### Statistical analysis

Continuous variables were analyzed with the Mann–Whitney U test, while dichotomous variables were analyzed with Fisher’s exact test. Progression-free survival (PFS) was defined as the time from the start of anti-PD-1 monotherapy to radiographic or clinical disease progression or death. OS was measured from the beginning of anti-PD-1 monotherapy to death from any cause. PFS and OS curves were generated using the Kaplan–Meier method and compared with the log-rank test. To minimize immortal time bias associated with the occurrence of pneumonitis, a 90-day landmark analysis was performed to evaluate the impact of ICI-induced pneumonitis on overall survival. Patients who died within 90 days of treatment were excluded from this analysis. Univariate and multivariate Cox proportional hazards models identified prognostic factors for OS, calculating hazard ratios (HRs) with 95% confidence intervals (CIs). Additionally, a subgroup analysis was performed among patients with available pulmonary function test data obtained within 6 months prior to anti-PD-1 monotherapy initiation. All P values were two-sided, with < 0.05 indicating statistical significance. Analyses were performed using EZR, a graphical user interface for R (The R Foundation for Statistical Computing, Vienna, Austria) [17]. The analysis cutoff date was May 15, 2024.

## Results

### Patients’ characteristics

This study included 200 patients with NSCLC treated with anti-PD-1 antibody monotherapy. Patient characteristics are detailed in Table 1. The pre-existing ILD group was significantly older, had a higher proportion of males, and more often had squamous cell carcinoma than the non-ILD group. No significant differences were found between the groups in smoking history, PS, clinical stage, PD-L1 levels, previous therapies, or the anti-PD-1 antibody used (nivolumab or pembrolizumab). All patients on pembrolizumab as first-line therapy had high PD-L1 expression, with this being more common in the ILD group. Regarding diagnostic CT criteria, 35 patients (17.5%) had pre-existing ILD, including three (1.5%) with UIP pattern, 14 (7.0%) with probable UIP, and 18 (9.0%) with indeterminate for UIP or an alternative diagnosis. The inter-observer agreement for the diagnosis and classification of pre-existing ILD showed a weighted Cohen’s kappa of 0.604 (95% CI, 0.499–0.710).

**Table 1:** Patients’ characteristics

Characteristics	Non-ILDN = 165	Pre-existing ILDN = 35	p
Age, years			0.0354
<65	71 (43.0)	8 (22.9)	
≥65	94 (57.0)	27 (77.1)	
Median (range)	66 (32–83)	69.0 (46–87)	
Sex			0.0194
Male	127 (77.0)	33 (94.3)	
Female	38 (23.0)	2 (5.7)	
Smoking status			0.134
Current/former smoker	145 (87.9)	34 (97.1)	
Never smoked	20 (12.1)	1 (2.9)	
Performance status			0.406
0	60 (36.4)	17 (48.6)	
1	93 (56.4)	16 (45.7)	
2	12 (7.3)	2 (5.7)	
Histology			0.0255
SQ	43 (26.1)	16 (45.7)	
Non-SQ	122 (73.9)	19 (54.3)	
Clinical stage			0.369
Stage III–IV	104 (63.0)	25 (71.4)	
Postoperative recurrence	30 (18.2)	7 (20.0)	
Postradiotherapy recurrence	31 (18.8)	3 (8.6)	
PD-L1 status			0.0992
<1%	15 (9.1)	0	
1%–49%	23 (13.9)	6 (17.1)	
≥50%	56 (33.9)	17(48.6)	
unknown	71 (43.0)	12 (34.3)	
Prior anticancer therapies			0.0546
0	38 (23.0)	14 (40.0)	
≥1	127 (77.0)	21(60.0)	
Anti-PD-1 antibodies			0.132
Nivolumab	100 (60.6)	16 (45.7)	
Pembrolizumab	65(39.4)	19 (54.3)	

*SQ* Squamous cell carcinoma, *PD-1* Programmed death 1, *PD-L1* Programmed death-ligand 1, *ILD* Interstitial lung disease

In addition, a subgroup analysis was conducted among patients with pulmonary function test data (*N* = 60). The baseline characteristics of this subgroup are summarized in Supplementary Table 1.

### Incidence of pneumonitis

Among all patients, 17.0% experienced ICI-induced pneumonitis, with 4.5% classified as grade ≥ 3. In the non-ILD group, 12.1% developed ICI-induced pneumonitis, with 3.6% being grade ≥ 3, whereas in the pre-existing ILD group, 40.0% had ICI-induced pneumonitis, with 8.6% at grade ≥ 3. This indicates a significantly higher rate of ICI-induced pneumonitis in the pre-existing ILD group (*P* < 0.001). The incidence of grade ≥ 3 pneumonitis did not differ significantly between the groups (*P* = 0.194). One case of grade 5 ICI-induced pneumonitis was observed in the pre-existing ILD group. Among patients who developed pneumonitis, the median time to onset was 62.5 days (interquartile range [IQR], 27.3–133.0) overall, 82.5 days (IQR, 28.0–138.3) in patients without pre-existing ILD and 41.0 days (IQR, 23.3–68.5) in those with pre-existing ILD (*P* = 0.172).

Among patients who developed pneumonitis, subsequent treatment was administered to 11 of 14 patients (78.6%) in the pre-existing ILD group and 11 of 20 patients (55.0%) in the non-ILD group (*P* = 0.275). Recurrent pneumonitis after subsequent treatment occurred in 3 of 11 patients (27.3%) in the pre-existing ILD group and 4 of 11 patients (36.4%) in the non-ILD group (*P* = 1.00). According to the severity of initial pneumonitis, patients with grade ≥ 3 pneumonitis were significantly less likely to receive subsequent treatment than those with grade 1–2 pneumonitis; subsequent treatment was administered to 2 of 9 patients (22.2%) and 20 of 25 patients (80.0%), respectively (*P* = 0.0037). Details of subsequent treatments are provided in Supplementary Table 2.

As an additional analysis, we evaluated the association between pulmonary function parameters—diffusing capacity of the lung for carbon monoxide (DLCO), forced expiratory volume in 1 s (FEV1), and forced vital capacity (FVC)—and ICI-induced pneumonitis development (DLCO ≥ 80% vs. <80%, %FEV1 ≥ 70% vs. <70%, and %FVC ≥ 80% vs. <80%) (Supplementary Table 3). Patients with %FEV1 < 70% showed a significantly higher incidence of ICI-induced pneumonitis compared with those with %FEV1 ≥ 70% (42.9% vs. 4.5%, *P* = 0.0016). Similarly, patients with %FVC < 80% had a higher incidence of ICI-induced pneumonitis than those with %FVC ≥ 80% (33.3% vs. 7.0%, *P* = 0.0218). In contrast, no significant association was observed between DLCO (< 80% vs. ≥80%) and ICI-induced pneumonitis development (16.7% vs. 13.0%, *P* = 0.665).

### Overall outcomes and comparison by ILD status

Overall, the response rate was 32.5%, with a median PFS of 4.6 months (95% CI, 3.1–5.8), and a median OS of 20.9 months (95% CI, 17.0–24.4) (Supplementary Fig. 1). The 5-year PFS rate was 10.7% (95% CI, 6.8–15.5), and the 5-year OS rate was 24.3% (95% CI, 18.6–30.4).

**Fig. 1:**
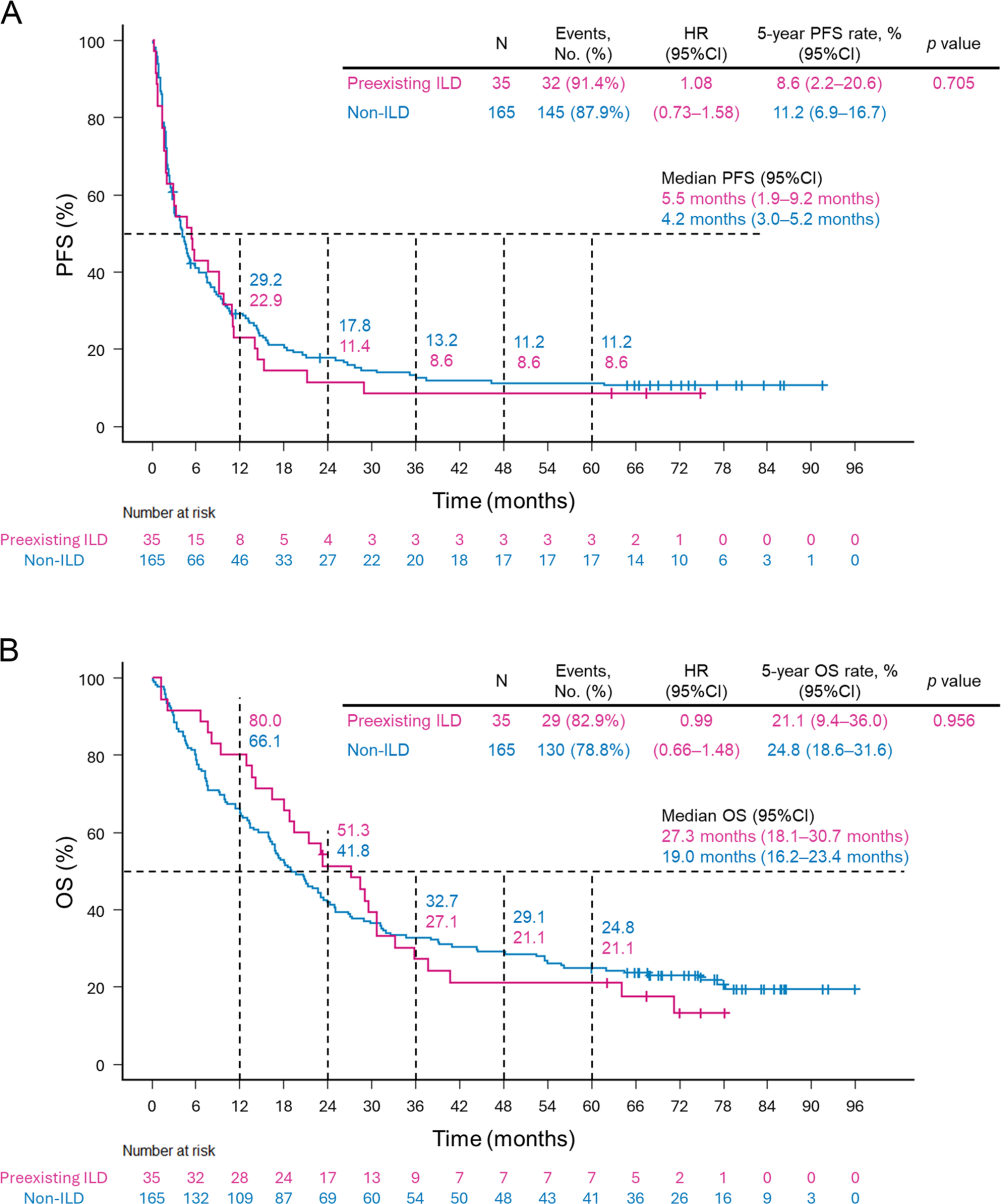
Kaplan–Meier curves showing (**A**) progression-free survival and (**B**) overall survival based on pre-existing ILD status. ILD, interstitial lung disease

When stratified by pre-existing ILD status, no significant differences were observed. The response rate was 34.3% in the ILD group and 32.1% in the non-ILD group (*P* = 0.844). The median PFS was 5.5 months (95% CI, 1.9–9.2) for the ILD group and 4.2 months (95% CI, 3.0–5.2) for the non-ILD group, with 5-year PFS rates of 8.6% and 11.2%, respectively (HR = 1.08; 95% CI, 0.73–1.58, *p* = 0.705) (Fig. 1A). The median OS was 27.3 months (95% CI, 18.1–30.7) for the ILD group and 19.0 months (95% CI, 16.2–23.4) for non-ILD, with 5-year OS rates of 21.1% and 24.8%, respectively (HR = 0.99; 95% CI, 0.66–1.48, *P* = 0.956) (Fig. 1B). OS analysis by ILD pattern, shown in Supplementary Fig. 2, indicated median OS of 28.5 months for UIP, 20.5 months for probable UIP, and 29.6 months for indeterminate UIP or other diagnoses. In a subgroup analysis of patients with available pulmonary function test data, baseline pulmonary function parameters (%FVC, %FEV1, and %DLCO) were not significantly associated with overall survival. The results of the univariable analysis are summarized in Supplementary Table 2.

### Comparison by line of therapy

Survival outcomes based on treatment line were also analyzed, revealing no significant differences between patients with and without pre-existing ILD. Among first-line treatment patients—who all had PD-L1 ≥ 50% and received pembrolizumab—pre-existing ILD patients had a median OS of 25.5 months (95% CI, 2.2–NA), and non-ILD patients 44.3 months (95% CI, 21.1–NA), with 5-year OS rates of 28.6% and 50.0%, respectively (HR = 1.84; 95% CI, 0.85–3.96, *P* = 0.115) (Supplementary Fig. 3A). In second-line or later treatment cases, pre-existing ILD patients had a median OS of 27.3 months (95% CI, 18.1–33.1), compared to 16.9 months (95% CI, 10.3–21.1) for non-ILD patients (HR = 0.87; 95% CI, 0.53–1.41, *P* = 0.561) (Supplementary Fig. 3B).

### Impact of pneumonitis on overall survival

**Fig. 2:**
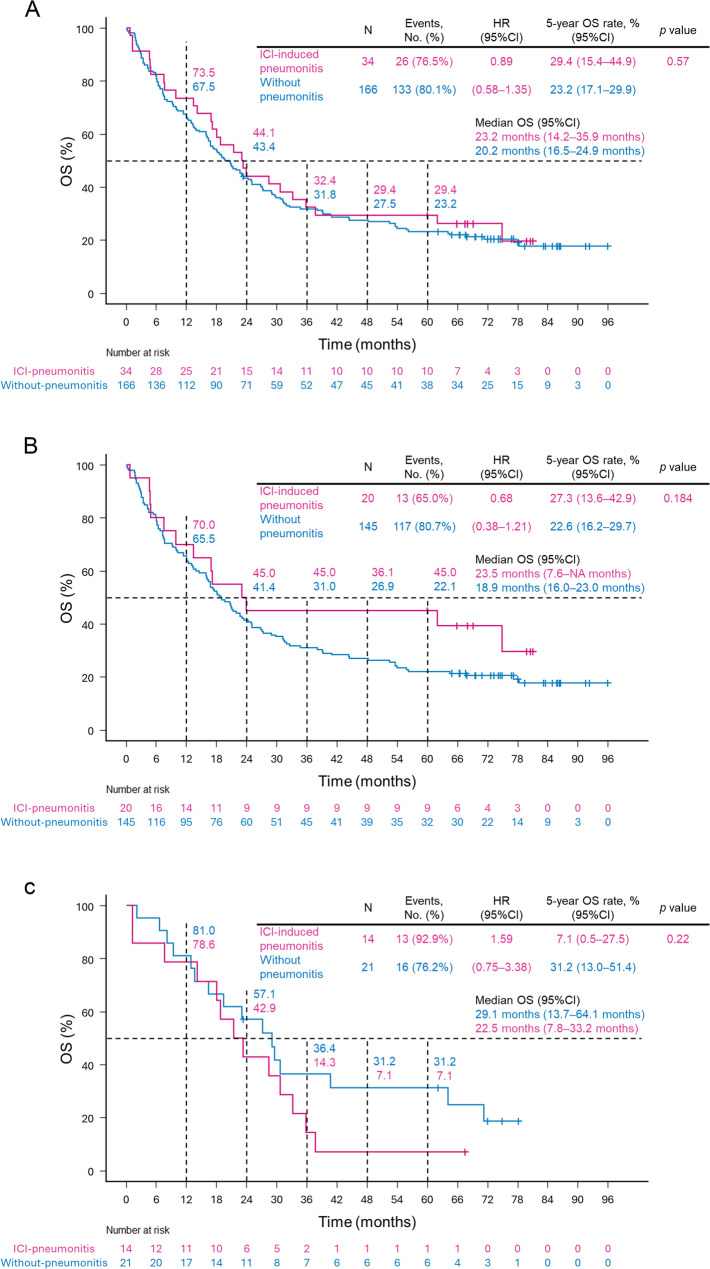
Kaplan–Meier survival curves showing overall survival based on developing ICI-induced pneumonitis (**A**) for all patients, (**B**) for patients without ILD, and (**C**) for patients with pre-existing ILD. ILD, interstitial lung disease; ICI, immune checkpoint inhibitors

The analysis assessed how ICI-induced pneumonitis influences OS, to determine whether developing pneumonitis impacts patient outcomes. Results indicated no statistically significant difference in OS between patients with and without ICI-induced pneumonitis; median OS was 23.2 months (95% CI, 14.2–35.9) for those with pneumonitis and 20.2 months (95% CI, 16.5–24.9) for those without (HR = 0.89; 95% CI, 0.58–1.35, *P* = 0.57) (Fig. 2A). Comparing OS in non-ILD cases with and without pneumonitis also showed no significant difference; median OS was 23.5 months (95% CI, 7.6–NA) with pneumonitis and 18.9 months (95% CI, 16.0–23.0) without (HR = 0.68; 95% CI, 0.38–1.21, *P* = 0.184) (Fig. 2B). In cases with pre-existing ILD, no significant OS difference was observed; median OS was 22.5 months (95% CI, 7.8–33.2) with pneumonitis and 29.1 months (95% CI, 13.7–64.1) without (HR = 1.59; 95% CI, 0.75–3.38, *P* = 0.22) (Fig. 2C).

### A landmark analysis at 90 days

**Fig. 3:**
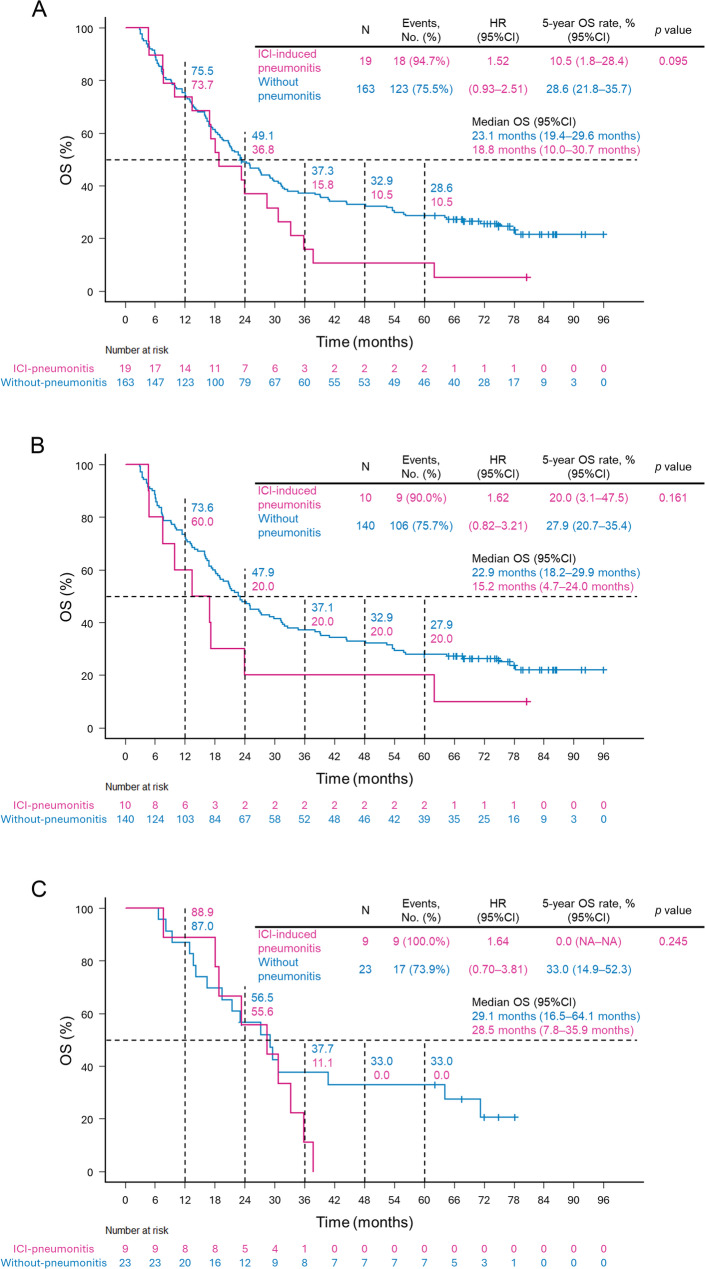
Kaplan–Meier survival curves in a 90-day landmark analysis show: (**A**) all patients included, (**B**) patients without ILD, and (**C**) patients with pre-existing ILD. ILD, interstitial lung disease

A landmark analysis at 90 days revealed no statistically significant difference in OS between patients with and without ICI-induced pneumonitis (HR = 1.52; 95% CI, 0.93–2.51, *p* = 0.095) (Fig. 3A). Similarly, no significant OS difference was seen between non-ILD and pre-existing ILD groups (non-ILD, HR = 1.62; 95% CI, 0.82–3.21, *P* = 0.161; pre-existing ILD, HR = 1.64; 95% CI, 0.70–3.81, *p* = 0.245) (Fig. 3B 3C).

### Multivariable Analysis of Overall Survival

Table 2 presents the results of the multivariable Cox proportional regression analysis for OS. PS 2 was significantly linked to shorter OS (HR = 3.82; 95% CI, 2.16–6.76, *P* < 0.001). In contrast, patients who received first-line treatment showed a notable improvement in OS compared to those treated with second-line or later regimens (HR = 0.44; 95% CI, 0.21–0.67, *P* < 0.001). Additionally, squamous cell carcinoma histology was associated with poorer OS (HR = 1.42; 95% CI, 1.00–1.99, *P* = 0.047). Variables such as age, sex, smoking status, and pre-existing ILD did not show significant associations.

**Table 2:** Multivariable analysis of overall survival

Parameter	Category	HR	(95% CI)	p
Age, years	≥65 vs. <65	1.27	(0.91–1.77)	0.17
Sex	Female vs. male	1.00	(0.65–1.53)	0.99
Smoking status	Smoker vs. never smoked	0.65	(0.38–1.14)	0.14
Performance status	≥2 vs. 0–1	3.82	(2.16–6.75)	<0.001
Line of treatment	First vs. second or later	0.44	(0.29–0.67)	<0.001
Histology	SQ vs. non-SQ	1.41	(1.00–1.99)	0.047
Pre-existing ILD	Yes vs. no	1.06	(0.69–1.61)	0.79

*ILD* Interstitial lung disease, *SQ* Squamous cell carcinoma, *HR* Hazard ratio, *CI* Confidence interval

## Discussion

To our knowledge, this is the first study to assess the 5-year efficacy and safety of anti-PD-1 antibody therapy in patients with NSCLC and pre-existing ILD. The group with pre-existing ILD showed a high rate of pneumonitis, a 5-year OS in the low-20% range, and a hazard ratio near one, suggesting no significant difference compared to patients without ILD.

Patients with pre-existing ILD have mostly been excluded from clinical trials of ICIs, resulting in limited data from intervention studies. Several single-arm phase II trials of nivolumab and atezolizumab have been conducted in patients with NSCLC and pre-existing ILD. However, data remain scarce, with only short-term efficacy and safety outcomes reported in these studies [14, 18]. The incidence of pneumonitis was notably higher in patients with pre-existing ILD compared to those without (40.0% versus 12.1%). Yet, there was no significant difference in PFS or OS between the groups. These findings suggest that the presence of pre-existing ILD does not significantly affect the efficacy of ICIs. Therefore, when administering ICIs to patients with NSCLC and ILD, careful management of the elevated pneumonitis risk is essential. Although the risk of ICI-induced pneumonitis is increased, our study recorded only one death due to ICI-induced pneumonitis (2.9%) among patients with pre-existing ILD. The OS rate was comparable to that of patients without ILD, indicating that the potential survival benefits of ICI therapy remain accessible to this group. While the transition rate to subsequent therapy did not differ between patients with and without ILD, patients with grade ≥ 3 pneumonitis were significantly less likely to receive subsequent treatment than those with grade 1–2 pneumonitis, suggesting that pneumonitis severity rather than pre-existing ILD influenced the feasibility of subsequent therapy.

No significant difference in OS was observed between patients with and without ICI-induced pneumonitis. A subsequent landmark analysis at 90 days examined the impact of early-onset pneumonitis on prognosis. No statistically significant difference in OS was observed between the two groups in the landmark analysis. The 90-day landmark was chosen because most cases of ICI-induced pneumonitis in NSCLC occur within 8–12 weeks of starting treatment, so this period is appropriate for evaluating OS more accurately [15, 19]. In a prior retrospective cohort study, over 60% of cases happened within 3 months of starting PD-1 inhibitor therapy, confirming the clinical relevance of this threshold [14]. A major limitation of landmark analysis in our cohort is the limited number of patients who developed pneumonitis before the 90-day landmark. Therefore, the statistical power may have been insufficient to detect a significant difference in survival. Although several reports indicate that early-onset immune-related adverse events are associated with a favorable prognosis, most reports suggest the opposite when it comes to pneumonitis [20–22]. Multiple reports suggest that patients with NSCLC who develop early-onset ICI-induced pneumonitis tend to have lower survival rates. The findings of this study align with those of earlier reports [23–26].

This study showed that the 5-year survival rate for patients with early-onset pneumonitis within 90 days was 10.5%. While this suggests a relatively poor prognosis for these patients, it is essential to note that before the advent of ICIs, the 5-year survival rate for patients with NSCLC ineligible for targeted therapy of driver mutations was under 5% [27–29]. The higher risk of ICI-induced pneumonitis should not lead to avoiding ICI therapy altogether. This is because patients with pre-existing ILD still experience survival benefits from ICI treatment, showing that, despite the increased risk, ICI remains a feasible and potentially advantageous option for them.

In the multivariable Cox proportional hazards regression analyses, poor PS and squamous cell carcinoma histology were independently linked to worse OS. Conversely, first-line treatment was significantly associated with improved prognosis. Several studies have indicated that PS serves as a prognostic factor in patients undergoing ICI [30, 31]. The improved prognosis of first-line treatment was primarily due to all cases having high PD-L1 expression, as required for insurance coverage at that time. This aligns with earlier studies showing that cases with PD-L1 expression of 50% or higher in first-line treatment tend to have better outcomes than cases with varying PD-L1 levels in later treatments [4, 13]. PS and line of therapy were similarly distributed between patients with and without pre-existing ILD, suggesting no major imbalance in these clinical characteristics. However, among patients with available PD-L1 data, no cases with pre-existing ILD and PD-L1 expression < 1% were observed. This may reflect a potential selection bias, whereby patients with both pre-existing ILD and low PD-L1 expression were less likely to receive ICI therapy in clinical practice. This study also identified squamous cell carcinoma as a poor prognostic factor. Similarly, previous reports have indicated that squamous cell carcinoma predicts poorer outcomes for ICI treatment [30]. Squamous cell carcinoma is frequently linked to smokers, and the high rate of comorbid conditions like heart disease can negatively impact prognosis [32].

Our study had several limitations. First, it was a retrospective study conducted at a single center with a small number of cases. Second, PD-L1 immunostaining became approved for diagnosis in Japan only in 2017, so many cases lacked this data prior to that year. Third, we used a landmark analysis at 90 days, which excluded patients who died within that period. Fourth, only three cases involved UIP pattern with honeycomb lungs, making it difficult to analyze ICI use in such instances. Despite these constraints, our findings offer valuable insights into the feasibility and clinical benefits of anti-PD-1 antibodies in this high-risk patient group over more than five years follow-up. Fifth, the diagnosis and classification of pre-existing ILD were performed by pulmonologists without review by a thoracic radiologist. However, the inter-observer agreement between the two pulmonologists was moderate to substantial (weighted Cohen’s kappa = 0.604), suggesting acceptable consistency in ILD classification. Nevertheless, the absence of radiologist review may have introduced classification bias.

## Conclusions

Our findings suggest that anti-PD-1 antibodies are effective and safe over the long term in patients with NSCLC and pre-existing ILD. Although, as noted earlier, patients with pre-existing ILD experienced a high rate of ICI-induced pneumonitis, their long-term outlook was similar to those without ILD. These findings offer valuable insights for determining the use of immune checkpoint inhibitors in patients with NSCLC and pre-existing ILD.

## Supplementary Information

Supplementary Material 1.

## Data Availability

The datasets used and/or analyzed during the current study are available from the corresponding author upon reasonable request.

## Abbreviations

PD-1: Anti-programmed death 1
ICI: Immune checkpoint inhibitor
ILD: Interstitial lung disease
NSCLC: Non-small cell lung cancer
PD-L1: Programmed death-ligand 1
OS: Overall survival
PS: Performance status
CT: Computed tomography
IHC: Immunohistochemistry
PFS: Progression-free survival
HR: Hazard ratio
CI: Confidence interval
