# Supplementary material for: Five-year prognostic impact of pre-existing interstitial lung disease in non-small cell lung cancer patients treated with anti-PD-1 monotherapy: a retrospective analysis
Source: BMC Cancer. 2026 Apr 25;26:735. doi: 10.1186/s12885-026-15989-1 (PMC13251138; doi:10.1186/s12885-026-15989-1)
Supplement: Supplementary file 1 — Supplementary Material 1. [file 12885_2026_15989_MOESM1_ESM.docx]

**Supplementary Figure 1.** Kaplan–Meier curves of (A) progression-free and (B) overall survival for all study participants.


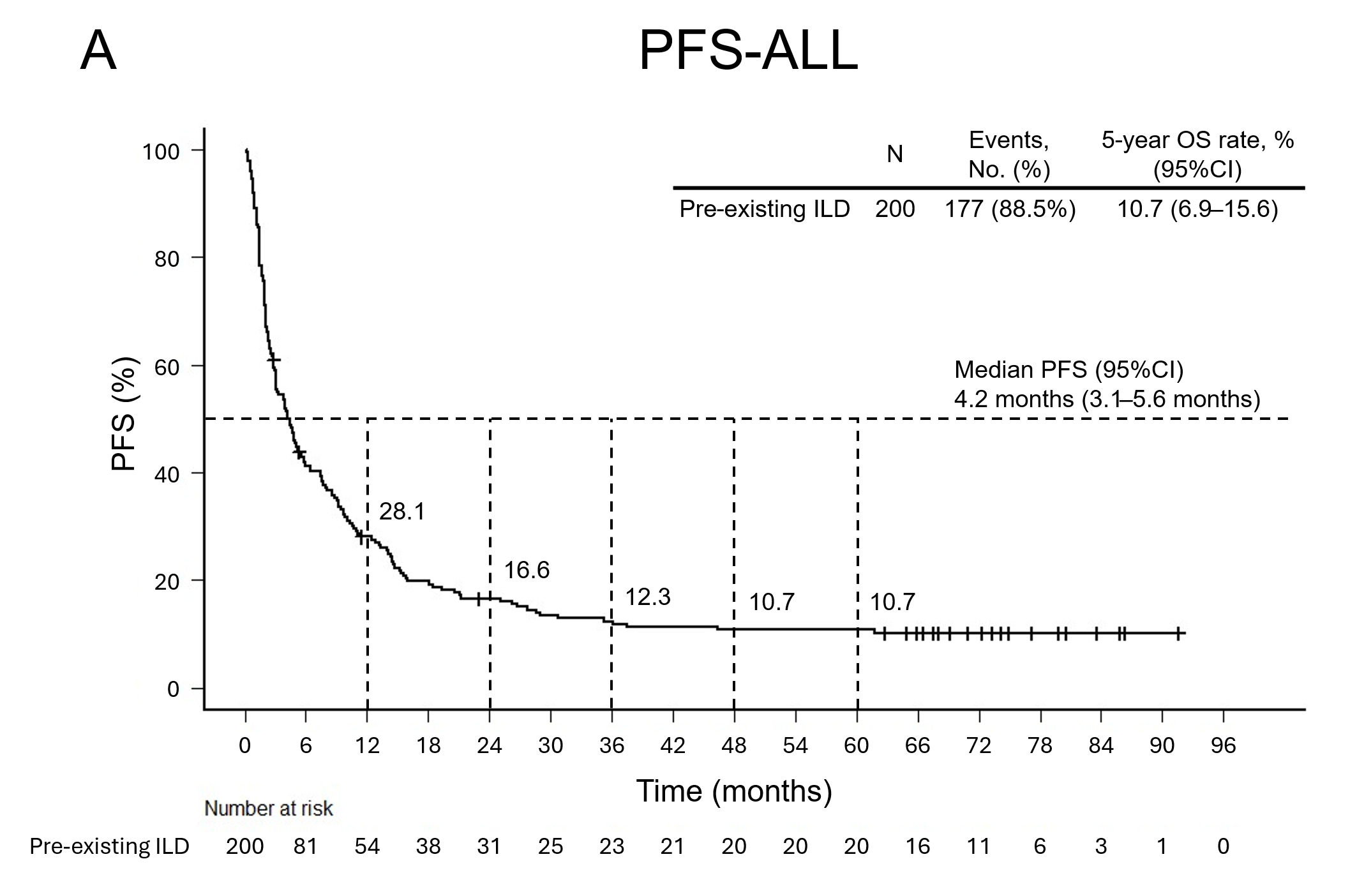


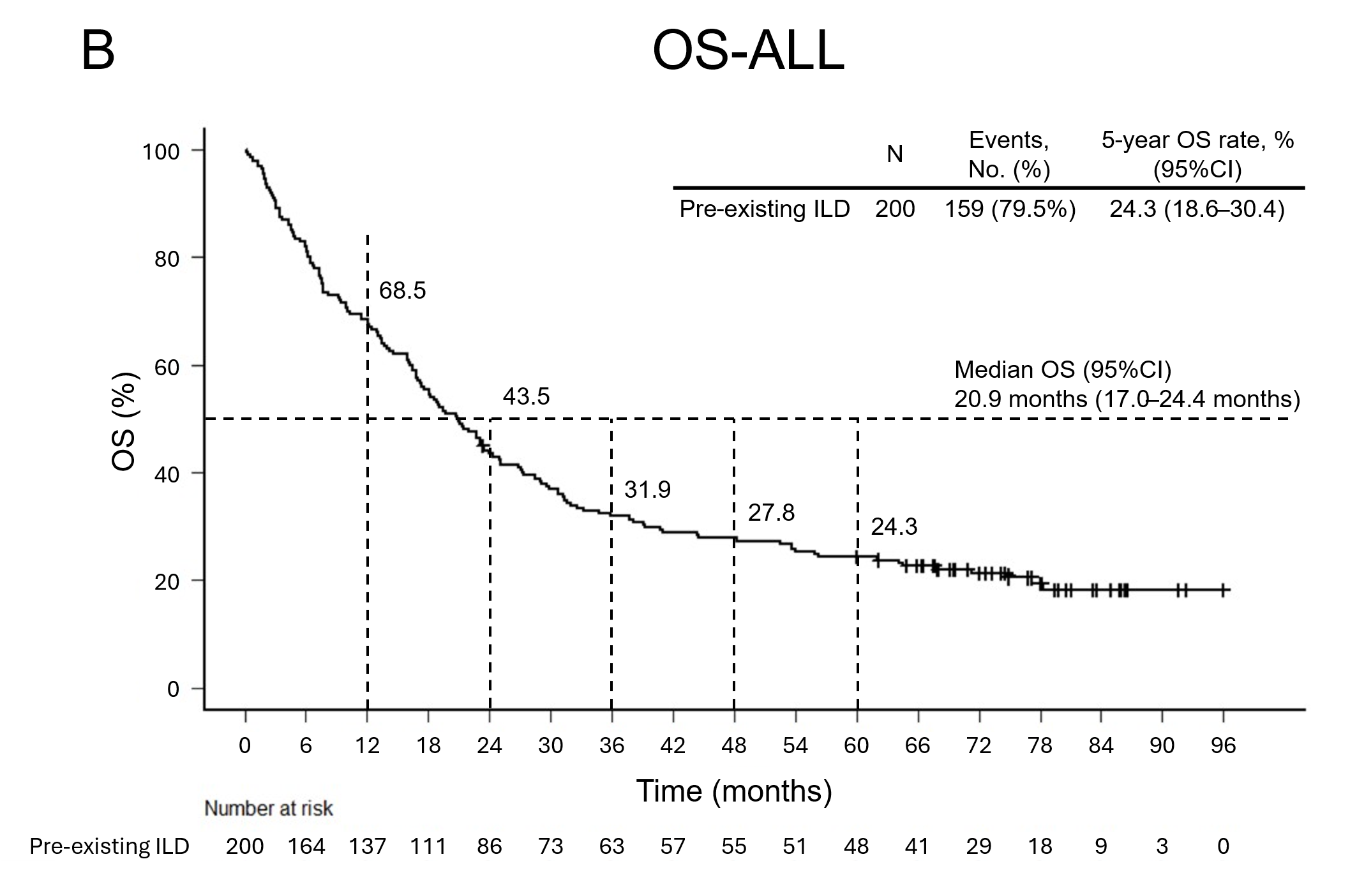


**Supplementary Figure 2.** Kaplan–Meier curves depicting overall survival relative to prior ILD status for patients who developed ICI-induced pneumonitis. ILD: interstitial lung disease. ILD, interstitial lung disease; ICI, immune checkpoint inhibitor


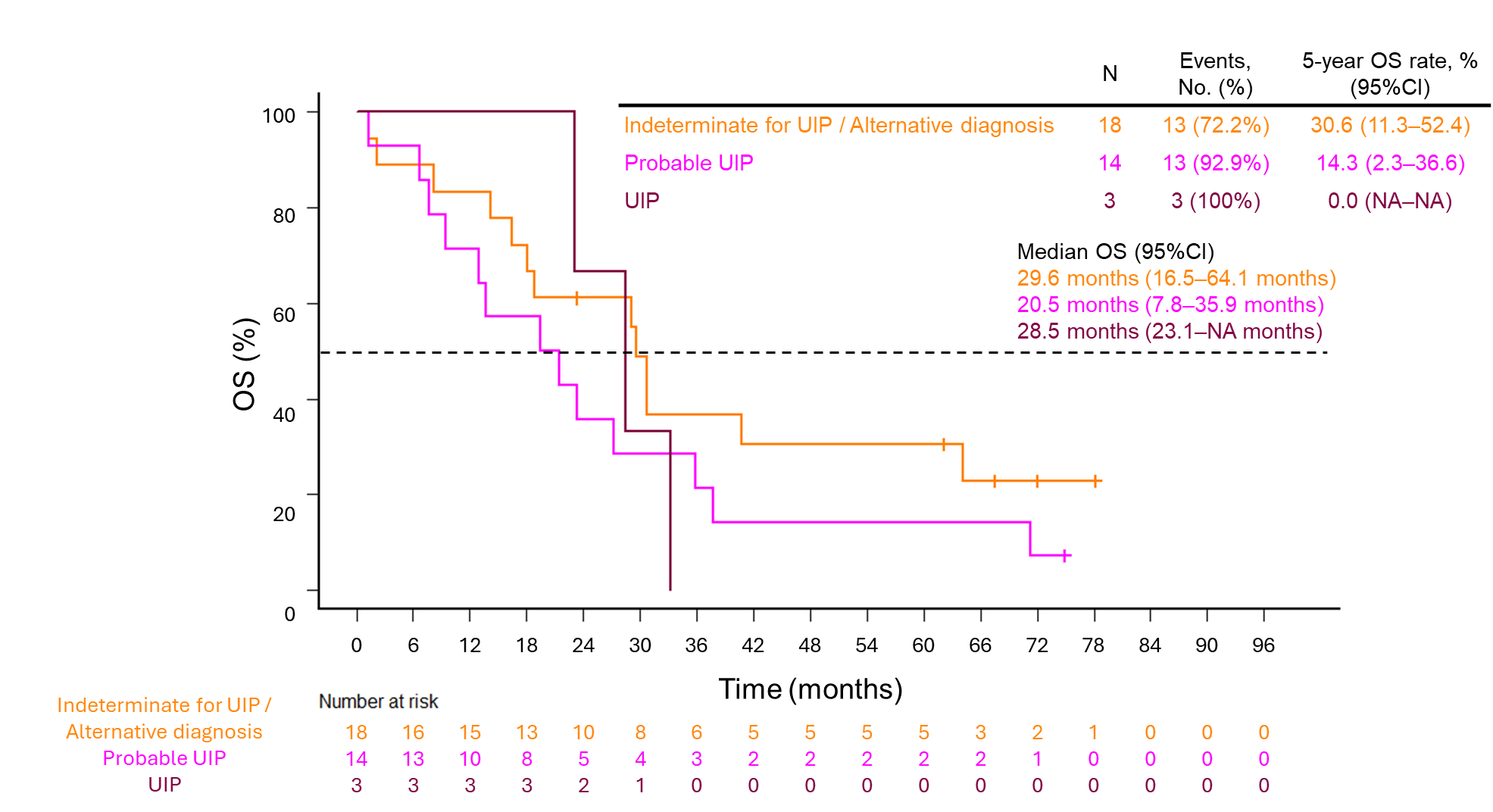


**Supplementary Figure 3.** Kaplan–Meier curves of overall survival corresponding to the ICI therapy line, (A) First-line and (B) second-line or later treatments are depicted. ICI, immune checkpoint inhibitor.


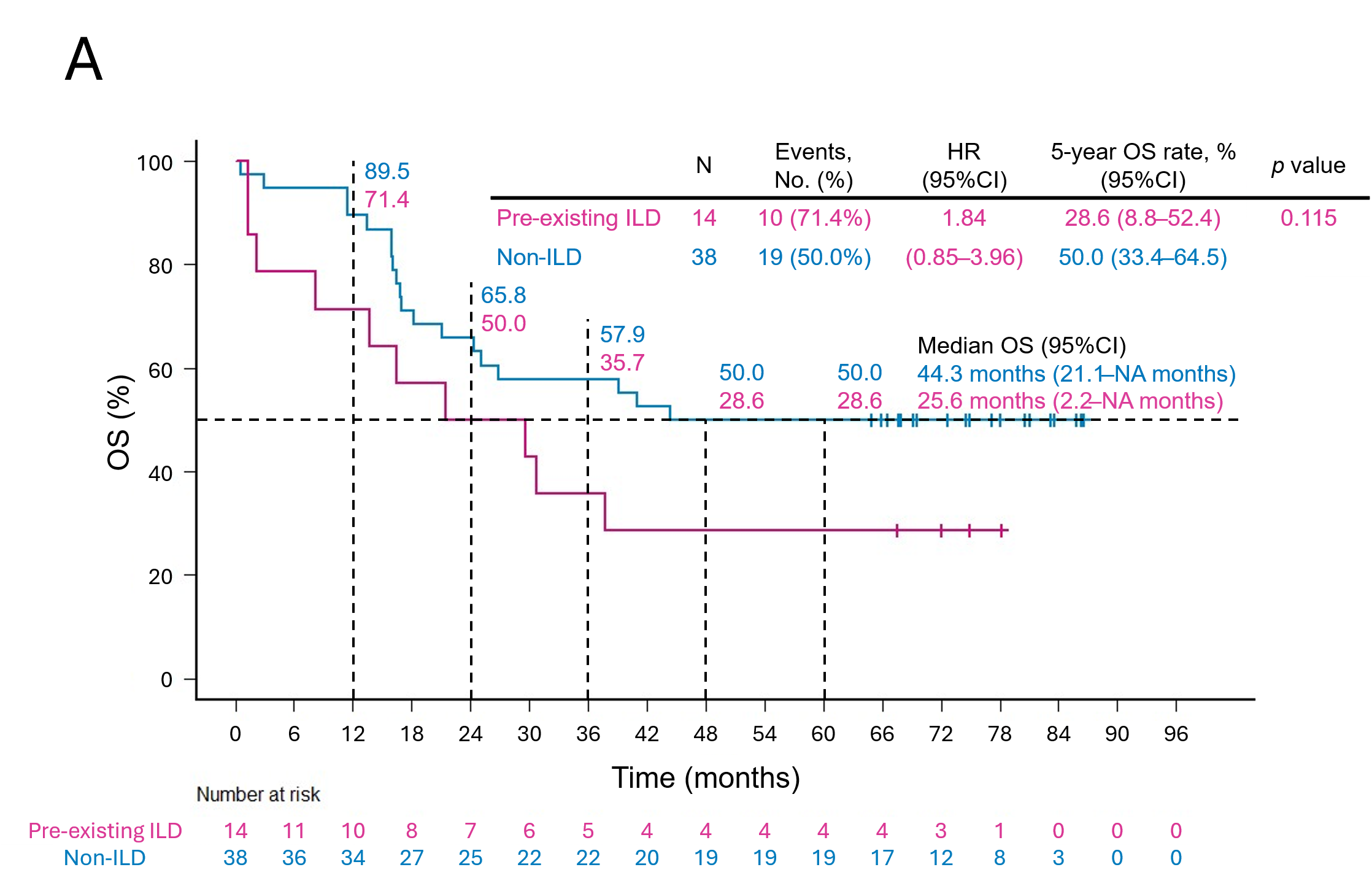


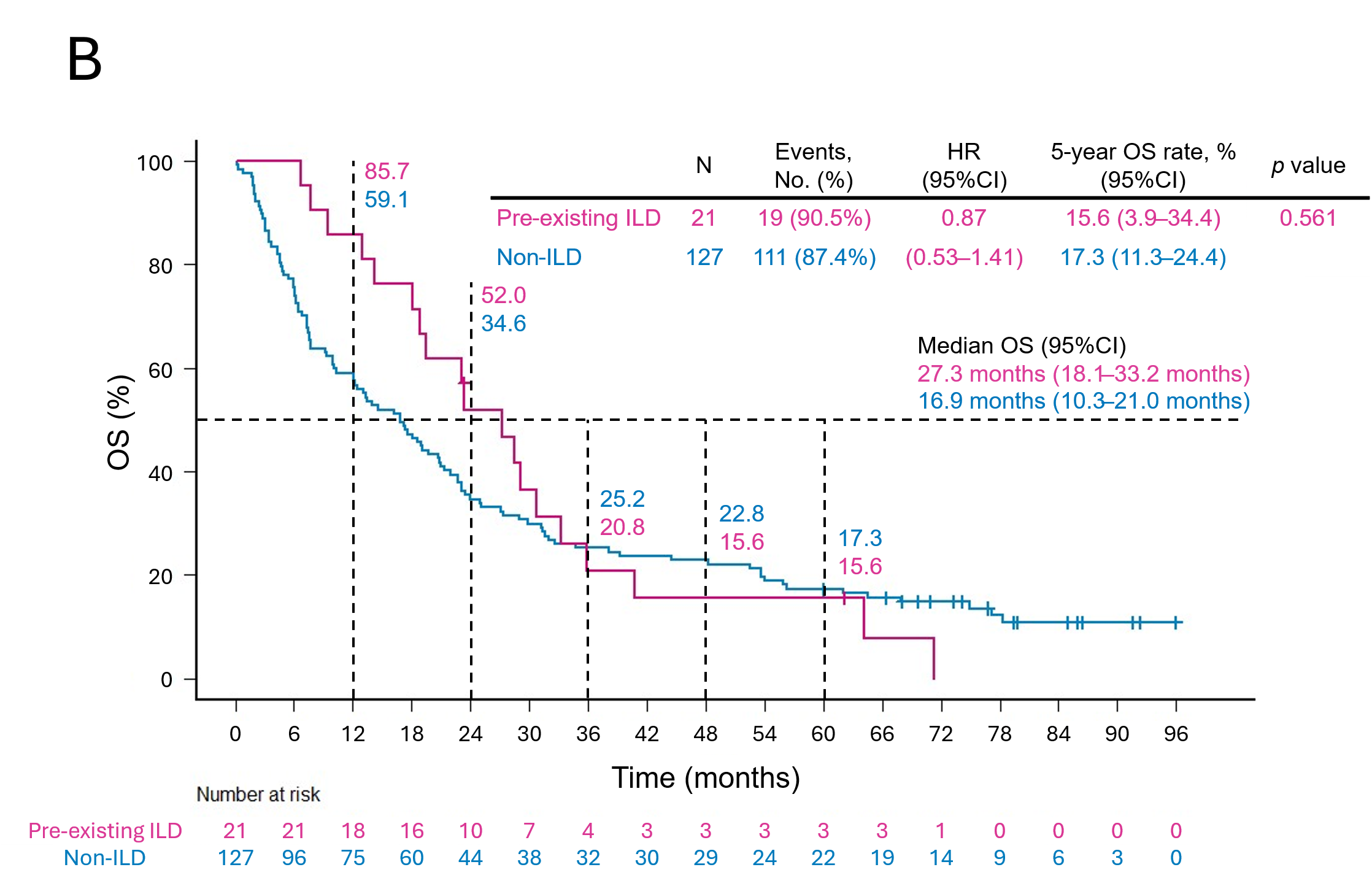


| **Supplementary Table 1.**  **Baseline characteristics of patients with available pulmonary function test data** | | | |  |
| --- | --- | --- | --- | --- |
| Characteristics | Non-ILD  N = 47 | Pre-existing ILD  N = 13 | *P* |  |
| Age, years |  |  | 1.000 |  |
| <65 | 24 (51.1) | 7 (53.8) |  |  |
| ≥65 | 23 (48.9) | 6 (46.2) |  |  |
| Median (range) | | 64 (39–83) | 64 (46–87) |  |
| Sex |  |  | 0.100 |  |
| Male | 36 (76.6) | 13 (100) |  |  |
| Female | 11 (23.4) | 0 |  |  |
| Smoking status |  |  | 1.000 |  |
| Current/former smoker | 41 (87.2) | 12 (92.3) |  |  |
| Never smoked | 6 (12.8) | 1 (7.7) |  |  |
| Performance status |  |  | 0.419 |  |
| 0 | 20 (36.4) | 8 (61.5) |  |  |
| 1 | 24 (56.4) | 4 (30.8) |  |  |
| 2 | 3 (6.4) | 1 (7.7) |  |  |
| Histology |  |  | 0.488 |  |
| SQ | 12 (25.5) | 5 (38.5) |  |  |
| Non-SQ | 35 (74.5) | 8 (61.5) |  |  |
| Clinical stage |  |  | 0.400 |  |
| Stage III–IV | 39 (83.0) | 12 (92.3) |  |  |
| Postoperative recurrence | 2 (4.3) | 1 (7.7) |  |  |
| Postradiotherapy recurrence | 6 (12.8) | 0 |  |  |
| PD-L1 status |  |  | 0.697 |  |
| <1% | 4 (8.5) | 0 |  |  |
| 1%–49% | 4 (8.5) | 1 (7.7) |  |  |
| ≥50% | 30 (63.8) | 12 (92.3) |  |  |
| unknown | 9 (19.1) | 0 |  |  |
| Prior anticancer therapies |  |  | 0.056 |  |
| 0 | 22 (46.8) | 2 (15.4) |  |  |
| ≥1 | 25 (53.2) | 11 (84.6) |  |  |
| Anti-PD-1 antibodies |  |  | 0.153 |  |
| Nivolumab | 15 (31.9) | 1 (7.7) |  |  |
| Pembrolizumab | 32 (68.1) | 12 (92.3) |  |  |
| %FVC (%) |  |  | 1.000 |  |
| <80 | 8 (17.8) | 4 (30.8) |  |  |
| ≥80 | 37 (82.2) | 9 (69.2) |  |  |
| %FEV1 (%) |  |  | 1.000 |  |
| <70 | 11 (24.4) | 3 (23.1) |  |  |
| ≥70 | 34 (75.6) | 10 (76.9) |  |  |
| %DLCO (%) |  |  | 0.437 |  |
| <80 | 12 (26.7) | 3 (23.1) |  |  |
| ≥80 | 33 (73.3) | 10 (76.9) |  |  |
| SQ, squamous cell carcinoma; PD-1, programmed death 1; PD-L1, programmed death-ligand 1; ILD, interstitial lung disease; %FVC, percent predicted forced vital capacity; %FEV1, percent predicted forced expiratory volume in 1 second; %DLCO, percent predicted diffusing capacity of the lung for carbon monoxide | | | |  |

| **Supplementary Table 2. Details of subsequent treatments and recurrent pneumonitis after ICI-induced pneumonitis** | | | | | |  |
| --- | --- | --- | --- | --- | --- | --- |
| Group | ILD pattern | Initial  pneumonitis  grade | Subsequent treatment | Recurrent pneumonitis | Recurrent pneumonitis grade | |
| Pre-existing ILD | UIP | 3 | Atezolizumab | Yes | 2 | |
| Pre-existing ILD | UIP | 1 | Pembrolizumab rechallenge | No | — | |
| Pre-existing ILD | Probable | 1 | Nivolumab rechallenge | No | — | |
| Pre-existing ILD | Probable | 1 | Nivolumab rechallenge | No | — | |
| Pre-existing ILD | Probable | 1 | Atezolizumab | No | — | |
| Pre-existing ILD | Probable | 1 | Carboplatin + nab-PTX | No | — | |
| Pre-existing ILD | Probable | 1 | Docetaxel | No | — | |
| Pre-existing ILD | Indeterminate for UIP / alternative diagnosis | 2 | Carboplatin + pemetrexed | Yes | 2- | |
| Pre-existing ILD | Indeterminate for UIP / alternative diagnosis | 2 | Carboplatin + nab-PTX | No | — | |
| Pre-existing ILD | Indeterminate for UIP / alternative diagnosis | 1 | Atezolizumab | No | — | |
| Pre-existing ILD | Indeterminate for UIP / alternative diagnosis | 1 | Docetaxel + ramucirumab | Yes | 2 | |
| Non-ILD | None | 3 | Atezolizumab | Yes | 1 | |
| Non-ILD | None | 2 | Pembrolizumab rechallenge | No | — | |
| Non-ILD | None | 2 | Carboplatin + pemetrexed | Yes | 3 | |
| Non-ILD | None | 1 | Nivolumab rechallenge | Yes | 2 | |
| Non-ILD | None | 1 | Nivolumab rechallenge | No | — | |
| Non-ILD | None | 1 | Nivolumab rechallenge | No | — | |
| Non-ILD | None | 1 | Pembrolizumab rechallenge | Yes | 1 | |
| Non-ILD | None | 1 | Pembrolizumab rechallenge | No | — | |
| Non-ILD | None | 1 | Pembrolizumab rechallenge | No | — | |
| Non-ILD | None | 1 | Carboplatin + pemetrexed + bevacizumab | No | — | |
| Non-ILD | None | 1 | S-1 | No | — | |
| ILD, interstitial lung disease; UIP, usual interstitial pneumonia; ICI, immune checkpoint inhibitor; nab-paclitaxel, nanoparticle albumin-bound paclitaxel. | | | | | |  |

| **Supplementary Table 3. Univariable analysis of overall survival in patients with available pulmonary function test data** | | | | |  |
| --- | --- | --- | --- | --- | --- |
| Parameter | Category | HR | (95% CI) | *P* |  |
| Age, years | ≥65 vs. <65 | 2.61 | (1.34–5.09) | 0.005 | |
| Sex | Female vs. male | 0.91 | (0.40–2.07) | 0.82 | |
| Smoking status | Smoker vs. never smoked | 1.37 | (0.48–3.87) | 0.55 | |
| Performance status | ≥2 vs. 0–1 | 9.45 | (3.06–29.15) | <0.001 | |
| Line of treatment | First vs. second or later | 0.57 | (0.30–1.09) | 0.090 | |
| Histology | SQ vs. non-SQ | 2.07 | (1.06–4.06) | 0.033 | |
| Pre-existing ILD | Yes vs. no | 1.57 | (0.75–3.25) | 0.23 | |
| %FVC, % | ≥80 vs. <80 | 1.83 | (0.80–4.21) | 0.16 | |
| %FEV1, % | ≥70 vs. <70 | 1.00 | (0.45–2.19) | 1.00 | |
| %DLCO, % | ≥80 vs. <80 | 0.83 | (0.38–1.82) | 0.64 | |
| ILD, interstitial lung disease; SQ, squamous cell carcinoma; HR, hazard ratio; CI, confidence interval; %FVC, percent predicted forced vital capacity; %FEV1, percent predicted forced expiratory volume in 1 second; %DLCO, percent predicted diffusing capacity of the lung for carbon monoxide | | | | |  |
